# Obstetric complications in women with polycystic ovary syndrome: a systematic review and meta-analysis

**DOI:** 10.1186/1477-7827-11-56

**Published:** 2013-06-26

**Authors:** Jun Z Qin, Li H Pang, Mu J Li, Xiao J Fan, Ru D Huang, Hong Y Chen

**Affiliations:** 1 Department of Obstetrics and Gynecology, First Affiliated Hospital of Guangxi Medical University, Nanning, China; 2 Department of Prenatal Diagnosis Center, First Affiliated Hospital of Guangxi Medical University, Nanning, China; 3 Department of Reproductive Center, First Affiliated Hospital of Guangxi Medical University, Nanning, China

**Keywords:** Polycystic ovary syndrome, Pregnancy and neonatal complications, Meta-analysis

## Abstract

**Background:**

Polycystic ovary syndrome (PCOS) is a common endocrine disorder in women of childbearing age. The risk of pregnancy and neonatal complications in women with PCOS is debatable. In order to determine the risk of pregnancy and neonatal complications, evidence regarding these risks was examined.

**Methods:**

Literature searches were performed in the electronic databases MEDLINE, EMBASE, and CENTRAL based on the established strategy and eligible tries were included according to inclusion and exclusion criteria. A systematic literature review looking at rates of gestational diabetes mellitus (GDM), pregnancy-induced hypertension (PIH), preeclampsia, premature delivery, neonatal birth weight, caesarean section and admission to a neonatal intensive care unit (NICU) was conducted in women with PCOS. Pregnancy outcomes between women with PCOS versus controls were included. Sensitivity analyses were performed to determine the reliability of the available evidence and to validate the results. The study was performed with the approval of the ethics committee of the First Affiliated Hospital of Guangxi Medical University.

**Results:**

A total of 27studies, involving 4982 women with PCOS and 119692 controls were eligible for the meta-analysis. Women with PCOS demonstrated a significantly higher risk of developing GDM (OR3.43; 95% CI: 2.49–4.74), PIH (OR3.43; 95% CI: 2.49–4.74), preeclampsia (OR2.17; 95% CI: 1.91–2.46), preterm birth (OR1.93; 95%CI: 1.45–2.57), caesarean section (OR 1.74; 95% CI: 1.38–2.11) compared to controls. Their babies had a marginally significant lower birth weight (WMD −0.11g; 95%CI: -0.19 – -0.03), and higher risk of admission to NICU (OR 2.32; 95% CI: 1.40–3.85) compared to controls.

**Conclusions:**

Women with PCOS have increased risk of adverse pregnancy and neonatal complications. It is necessary to establish guidelines for supervision during pregnancy and parturition to prevent these complications.

## Background

Polycystic ovary syndrome (PCOS) is a common and complicated female endocrinopathy that estimated prevalence varies from 3%–20% depending on the diagnostic criteria used [1]. The most common features of PCOS are abnormal ovulation, clinical or laboratory indices of increased androgen levels, and polycystic ovaries on ultrasonography. Clinical manifestations of PCOS are menstrual irregularity (oligomenorrhea or amenorrhea), hirsutism, persistent acne, androgen dependent alopecia, abdominal obesity, hypertension and infertility [2]. Although the prevalence of PCOS and diversified clinical symptoms are known, the exact pathogenesis of PCOS is not fully recognized yet.

It is commonly believed that insulin resistance, hyperandrogenism and obesity play a significant role on the pathophysiologic process of PCOS [3,4]. Insulin resistance is universally accepted as one of the key biochemical features of PCOS supported by complementary hyperinsulinemia, and is associated with ovarian secretion disorder increasing the androgen production by theca cells that lead to hyperandrogenism [5,6]. Obesity, a characteristic of 60–80% of PCOS patients, has a malignant additive effect on features of PCOS such as insulin resistance, hyperandrogenism, infertility, hirsutism and pregnancy complications [7]. However, the definite phenotype of PCOS (different combinations of oligo/anovulation, hyperandrogenism, polycystic ovaries), as well as the extent of obesity in PCOS patients influences the variation of insulin resistance level [8,9]. Furthermore, the interaction of insulin resistance, hyperandrogenism and obesity results in an increased risk of diabetes mellitus type 2 (DM2), metabolic syndrome (MS), cardiovascular diseases (CVD), pregnancy loss and late pregnancy complications (preeclampsia, gestational diabetes). This indicates that PCOS is a chronic disease that impacts women across the lifespan [10].

Nowadays a growing body of evidence points to a high prevalence of pregnancy complications in PCOS women. As a result, PCOS is not only related to metabolic abnormalities, menstrual irregularity or infertility as previously reported, but becoming increasingly recognized the problems of gestational diabetes (GDM), pregnancy-induced hypertension, preeclampsia, premature delivery rate, neonatal birth weight, caesarean section rate, and rate and admission to an NICU, which are all considered to be adverse pregnancy outcomes of PCOS during pregnancy [11-13]. The elevated risk for adverse obstetric complications that was observed in women presenting PCOS varied widely depending on the different phenotypes and features of PCOS [14]. Women with PCOS tend to require ovulation induction or assisted reproductive technology (ART) in order to become pregnant due to oligo-ovulation or anovulation, this treatment for infertility often results in an evaluated rate of multiple births [15,16]. In order to explore the relationship between PCOS and pregnancy outcomes completely, the use of metformin, ovulation induction or ART must be taken into account.

There have been a number of relevant studies performed in order to illustrate incidences of pregnancy and neonatal complications. However, the results of these studies have often been inconsistent, and two previous meta-analyses published on this issue have been questioned for the statistical heterogeneity [17,18]. To derive a more precise estimation of the risks of obstetric complications in women with PCOS, a further meta-analysis with updated data should be made. Therefore, we conducted an updated meta-analysis using different statistical methods exist for combining the data, to reassess the risks of pregnancy and neonatal complications in women with PCOS versus controls. To the best of our knowledge, no meta-analysis with the use of sensitive analysis on this issue has ever appeared.

## Methods

### Search strategy

To select qualified studies, a search was performed in the electronic databases MEDLINE, EMBASE, and Cochrane Central Register of Controlled Trials (CENTRAL) from 1966 through July 2012, the search strategy was conducted depend on various combinations of the terms [‘Polycystic Ovary Syndrome’ (MeSH) OR Hyperandrogenism (MeSH)] AND [‘obstetric outcomes’ (MeSH) OR ‘Pregnancy Outcome’ (MeSH) OR ‘Pregnancy Complications’ (MeSH) OR ‘Diabetes Mellitus, Type 2’ (MeSH)OR PIH OR pre-eclampsia OR preterm labor OR GDM OR fetal outcome OR neonatal outcome] with no language limitation. And then a manual search of the abstracts from the major annual meetings in the field about Human Reproduction. The main search was completed independently by two reviewers (Lihong Pang and Junzhen Qin.). Discordance was settled by consultation of a third reviewer (Mujun Li).The inclusion and exclusion criteria are presented in Table 1.

**Table 1:** Inclusion and exclusion criteria in examination of studies

**Inclusion criteria**	**Exclusion criteria**
1. Diagnosis of PCOS accordance with to the NIH, Rotterdam or AES criteria.	1. Studies that included women with preexisting diabetes mellitus or Hypertension.
2. Use of women without PCOS as controls.	2. Metformin was used by the PCOS group after conception.
3. End points include GDM, PIH, PE, preterm, birth weight, caesarean section or admission to NICU.	3. Multiple pregnancy rate was significantly different in the two groups.

*NIH* National Institutes of Health [19], *AES* the Androgen Excess Society [20].

### Eligibility of relevant studies

**Figure 1:**
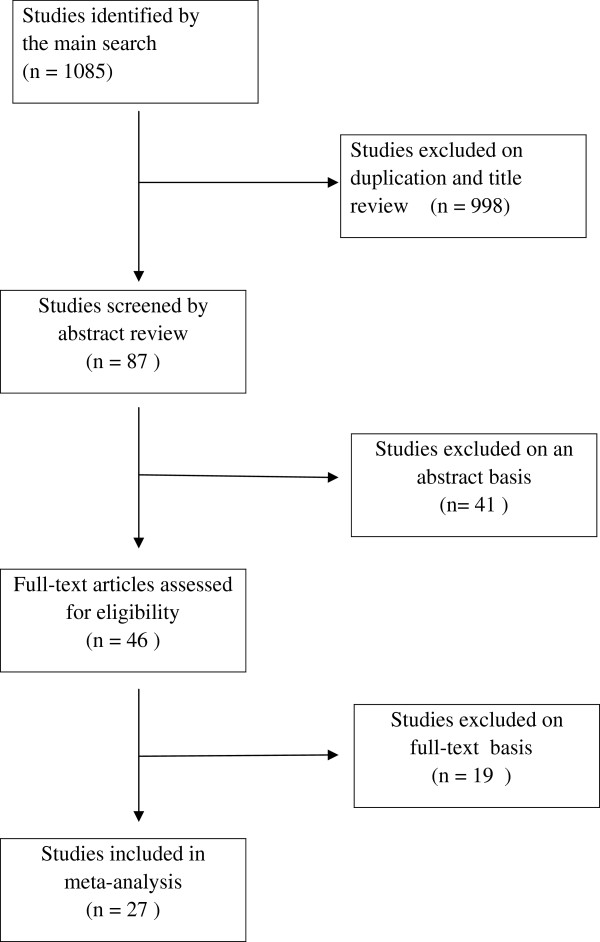
**Flow diagram of included studies for this meta**-**analysis.**

The main search strategy identified 1085 publications, 998 publications were excluded because of duplication or obviously irrelevance by title, then 41 articles were excluded on the basis of the abstract. Of the remaining 46 articles which were read in full by two reviewers independently in strict accordance with the described selection criteria. 19 articles were excluded because 6 lacked selection criteria, 3 did not evaluate the included outcomes, and 10 involved the use of metformin during pregnancy. Eventually, 27 studies were eligible for the meta-analysis (see Figure 1).

### Data extraction

Data extraction was conducted from all relevant studies independently by two reviewers. Information was classified summarized as follow: general characteristics (author, year of publication, study design, study center, study size and ratio of cases to controls), characteristics of the PCOS and control groups (method of conception, multiple pregnancies, and whether matched for age, body mass index or parity). Try best to communicate with the authors when data incomplete. Major characteristics are summed up in Table 2 ‘see Additional file 1: Table S1’.

**Table 2:** Main characteristics of included studies

**Study no.**	**Author**	**Year**	**Study design**	**Study center**	**Study size**	**No. of PCOS/control**	**Outcomes included**	**PCOS group**	**Control group**	**Multiple pregnancies**	**Notions**
**1**	Diamant [21]	1982	Retrospective	single center	72	33/39	PE, birthweight	Pregnant by ovulation induction	Anovulatory women pregnant by ovulation	Included,but equal Incidence	Matched for: age, similar parity
**2**	Levran [22]	1990	Retrospective	single center	171	76/95	GDM	Pregnant by CC,,hMG,or spontaneous	Normal pregnant Women	Not stated	Matched for age and BMI
**3**	Wortsman [23]	1991	Retrospective	multicenter	2359	53/2,306	GDM, birthweight	Pregnant by CC, hMG, dexamethasone, bromocriptine	Normal pregnant Women	Included,no significantly difference	Matched for age and BMI
**4**	Cardenas [24]	1996	Retrospective	single center	109	77/31	GDM, Birthweight	Pregnant by ovulation induction	Normal pregnant Women	Not included	matched for age and gravidity
**5**	Urman [25]	1997	Retrospective	single center	147	47/100	GDM,PIH, PE,preterm, NICU	Pregnant by CC,,hMG, dexamethasone,IVF	Normal pregnant Women	Not included	matched for age and gravidity, PCOS higher BMI
**6**	Lesser [26]	1997	Retrospective	single center	68	24/44	GDM	Pregnant by CC, hMG	infertility women pregnant by CC,hMG	Not stated	PCOS higher BMI
**7**	Fridstrom [27]	1999	Retrospective	single center	99	33/66	GDM,PIH,PE,birthweight, Cesarean section,NICU	Pregnant by IVF, ovulation Induction	Normal pregnant women	Included,,no significantly difference	matched for age and treatment
**8**	Radon [28]	1999	Retrospective	single center	88	22/66	GDM,PE, Birthweight	Pregnant by CC, hMG, IVF,or spontaneous	Normal pregnant Women	Not included	Matched for age and BMI
**9**	Kashyap [29]	2000	Retrospective	single center	49	22/27	PIH	Pregnant by hMG, ovulation Induction	Pregnant by hMG, IVF/IUI	Not stated	Similar BMI, age, parity
**10**	Vollenhoven [30]	2000	Retrospective	single center	132	60/72	GDM,PIH, preterm, birthweight, Cesarean section	Pregnant by ovulation induction	Normal pregnant Women	Included,no significantly difference	matched for age, BMI
**11**	Mikola [31]	2001	Retrospectiv	multicenter	836	80/728	GDM,PE, preterm, birthweight, Cesarean section	Pregnant by CC, gonadotrophins ,IVF	Normal pregnant Women	only singleton result included	PCOS higher BMI
**12**	Bjercke [11]	2002	Prospective	single center	407	52/355	GDM,PIH,PE,preterm, birthweight, Cesarean section, NICU	Pregnant by CC, hMG, IVF, IUI	Pregnant by ART	Not included	PCOS higher BMI
**13**	Haakova [32]	2003	Retrospective	Multicenter	132	66/66	GDM,PIH, preterm, birthweight, Cesarean section	Pregnant by ovulation Induction	Normal pregnant Women	Included,no significantly difference	Matched for age and BMI
**14**	Turhan [33]	2003	Retrospective	single center	174	38/136	GDM,PIH,PE,preterm, birthweight, Cesarean section,NICU	Not stated	Normal pregnant Women	Not included	matched for age
**15**	Weerakiet [34]	2004	Retrospective	single center	311	47/264	GDM,PIH,PE, preterm, birthweight, Cesarean section	Pregnant by CC, IVF, ovarian drilling	Normal pregnant Women	Not included	PCOS higher BMI
**16**	Sir-Petermann [35]	2005	Prospective	single center	227	47/180	GDM,PE, preterm, birthweight, NICU	Not stated	Normal pregnant Women	Not included	matched for age,BMI
**17**	Al-Ojaimi [36]	2006	Prospectiv	single center	513	134/479	GDM,PIH,PE, preterm, birthweight	treated with laparoscopic ovarian drilling	Normal pregnant Women	Not included	PCOS higher BMI
**18**	Hu [37]	2007	Prospective	single center	44	22/22	PIH, Birthweight	Spontaneous	Normal pregnant Women	Not included	matched for age,BMI, parity
**19**	Sir-Petermann [38]	2007	Prospective	single center	99	48/51	GDM,PIH	Not stated	Normal pregnant Women	Not stated	matched for age
**20**	Maliqueo [39]	2009	Prospective	single center	64	30/34	Birthweight	Spontaneous	Normal pregnant Women	Not included	PCOS higher BMI
**21**	Palomba [14]	2010	Prospective	single center	162	93/69	GDM,PIH,PE, preterm, Cesarean section	Spontaneous	Normal pregnant Women	Not included	matched for age, BMI and parity
**22**	Altieri [40]	2010	Retrospective	single center	174	15/159	GDM,PIH,PE, preterm, birthweight, Cesarean section	Spontaneous, ovulation induction, ART	Normal pregnant Women	Not included	matched for age, BMI and parity
**23**	Li [41]	2010	Prospective	single center	104	34/70	PE,preterm, birthweight	Pregnant by ART, Spontaneous	Normal pregnant Women	Not included	PCOS higher BMI
**24**	Roos [42]	2011	Prospective	single center	1195123	3787/ 1 191 336	GDM,PE ,preterm, Cesarean section	Pregnant by ART	Normal pregnant Women	Not included	PCOS higher BMI
**25**	Dmitrovic [43]	2011	Prospective	single center	34	17/17	GDM, Birthweight	Not stated	Not stated	Not included	results adjust for BMI
**26**	Han [44]	2011	Retrospective	Not stated	1339	336/1003	PIH,preterm	Pregnant by ovulation induction, ART	pregnant by ovulation induction, ART	Included,no significantly difference	matched by age, PCOS higher BMI
**27**	Reyes-Munoz [45]	2012	Retrospective	single center	104	52/52	GDM,PE, preterm, birthweight	Achieved pregnancy after OCs,CC	Normal pregnant Women	Not included	matched by age, parity, BMI

*GDM* gestational diabetes mellitus, *PIH* pregnancy-induced hypertension, *PE* preeclampsia, *NICU* admission to a neonatal intensive care unit, *ART* assisted reproductive technology, *IVF* In vitro fertilization, *CC* clomifene, *OC* Oral contraceptive. ‘See Additional file 1: Table S1’.

### Statistical analysis

odds ratios (OR) with 95% confidence intervals (CI) for dichotomous data, weighted mean difference (WMD) with 95% CI for Continuous data, and both combined with use of a fixed-effects or random-effects model, where appropriate. Heterogeneity between the results of different studies was detected byχ^2^ tests for significance (a P value of <0.1 was considered statistically significant) and I^2^ test (I^2^ <25%: insignificant heterogeneity, I^2^ >50%: significant heterogeneity). The Egger test was used for evaluating the degree of publication bias. To prove reliability of the available evidence and get convincing results, sensitivity analyses were manipulated with the exclusion of studies with borderline eligibility. Discordance among reviewers on studies with borderline eligibility was resolved by consensus. Subsequently, if the number of included studies were more than 10, univariate meta-regression analyses were performed on the effect of a study-level characteristic to guarantee explainable outcomes. Meta-analysis and meta-regression were conducted using Stata/SE 12.0 for Windows (StataCorp LP, College Station, USA).

## Results

A total of 27 studies, involving 4982 women with PCOS (4994 pregnancies) and 119692 controls (1,196,775 pregnancies), were eligible for the systematic review and meta-analysis. The smallest study size of included studies is 34 [43], the maximal number is up to 1195123 [42].

### Systematic review

Main characteristics of eligible studies are summarized in Table 2. All the studies mentioned age, BMI, as well as multiple pregnancies. The diagnostic standard of PCOS is accordant with NIH 1990 criteria, the Rotterdam 2003 criteria or the AES 2006 criteria. Most of the studies were retrospective in design, only 10 studies (37.0%) were prospective. A total of 3 studies (11.1%) were multicenter, 1 study did not state whether it was multi- or single center, and the rest were single center. The definitions of pregnancy and neonatal complications: gestational diabetes mellitus is mainly diagnosed with a 50–100 g oral glucose challenge test; pregnancy-induced hypertension depends on BP ≥ 140/90 mmHg without proteinuria at a gestational age of >20 weeks; preeclampsia according to criterion with BP ≥ 140/90 mmHg with proteinuria >0.3 g/24h /≥2 + albustick at a gestational age of >20 weeks; premature delivery (gestational age <37 weeks); birth weight (g).

It is noted that higher valid studies are designated when confounding variables (BMI, multiple pregnancies rate, and selection of controls) are managed in PCOS and control group. In 10 of 27 studies, the mean BMI of PCOS women was significantly higher than that of controls. Among the other 17 studies mean BMI was matched in the two groups. In 6 studies there was an equal incidence of multiple pregnancies in both study groups. In one study, the outcomes of multiple and single pregnancies were separate. In the remaining studies, only single pregnancy was recorded. Of the 27 studies, 21 selected women who became pregnant naturally (with no infertility treatments) as controls, 5 included women who had infertility treatments, and 1 did not describe the controls. Beyond that, method of conception was not mentioned for the PCOS group in 11.1% studies, whereas in the remaining 89.9% studies ovulation induction or assisted reproduction techniques (ART) were used for conception in PCOS patients.

Among the total studies, significantly increased risks of PCOS patients compared with controls were found in 11/21 studies GDM, 7/14 studies PIH, 5/15 studies preeclampsia, 1/14 studies preterm, 5 /19 studies birth weight, 3/ 10 studies caesarean section and 1/5 studies admission to an NICU. Consequently, the relationship between PCOS and obstetric complications seem not robust viewing from the above summative data. To evaluate the adverse risk of pregnancy and neonatal complications in women with PCOS, we have performed a systematic review and meta-analysis of the best available trials.

### Meta-analysis

#### Gestational diabetes mellitus

**Figure 2:**
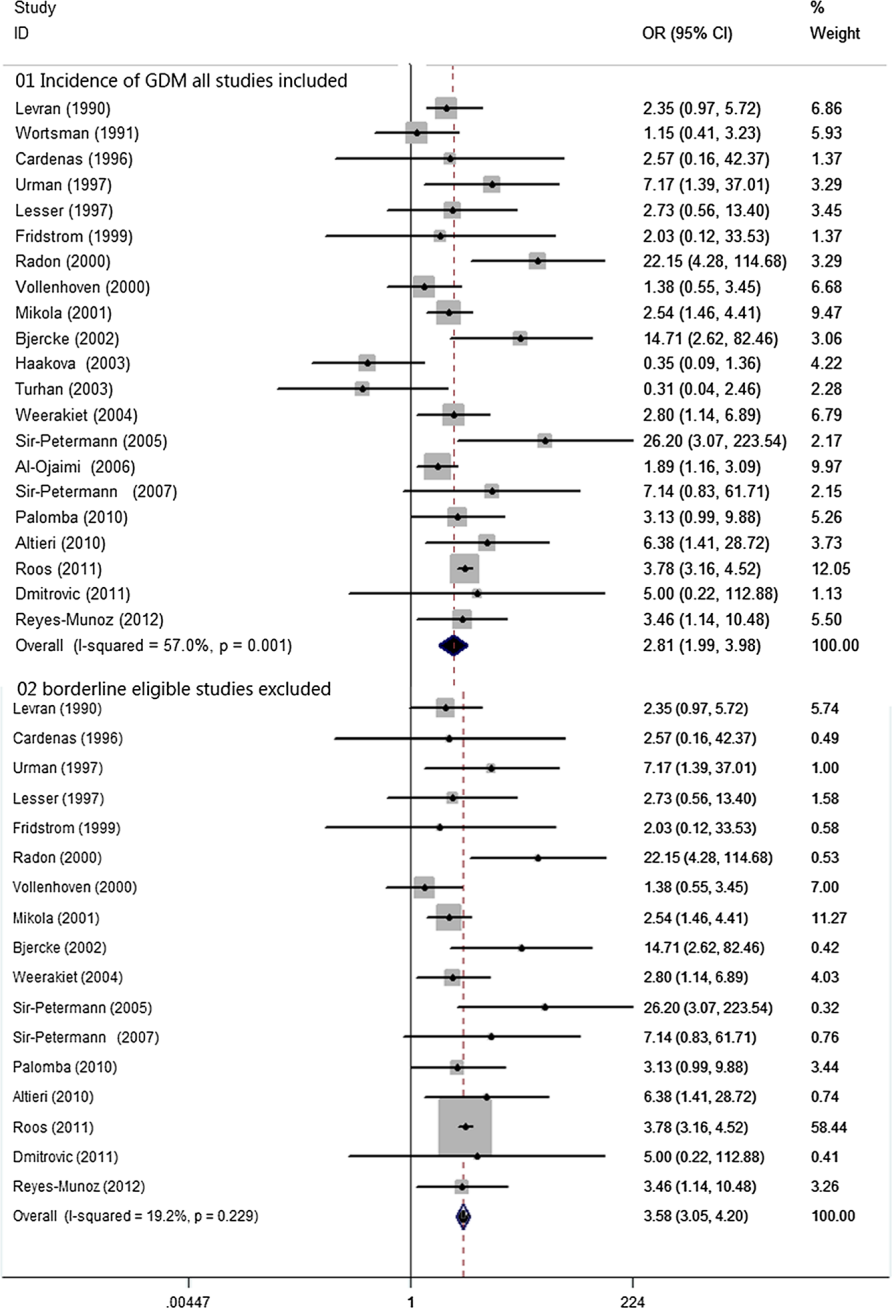
**OR for incidence of GDM in women with PCOS and controls. 01** Incidence of GDM all studies included. **02** borderline eligible studies excluded.

There were 21 studies, involving 4841 women with PCOS and 1196705 controls, eligible for the meta-analysis on the risk of development of GDM. Women with PCOS demonstrated a significantly elevated chance of developing GDM when compared with controls, yet with significant between-study heterogeneity [21 studies, random effects OR 2.81(95% CI: 1.99–3.98) heterogeneity χ^2^: P=0.001, I^2^= 57.0%; Figure 1]. Publication bias was insignificant using the Egger test (P=0.53). It was noted that, with the exclusion of 4 studies with borderline eligibility [23,32,33,36], the sensitivity analysis ‘see Additional file 2: Figure S1’, did substantially decrease in insignificant heterogeneity (χ^2^: P=0.229, I^2^= 19.2%), and the difference in the risk of development of GDM between women with PCOS and controls remained robust [17 studies, fixed effects OR 3.58 (95% CI: 3.05–4.20) Figure 2]. Meta-regression failed to provide evidence of a significant effect between outcome and study type (retrospective vs. prospective) (P=0.18) or BMI (P= 0.974).

### Pregnancy-induced hypertension

**Figure 3:**
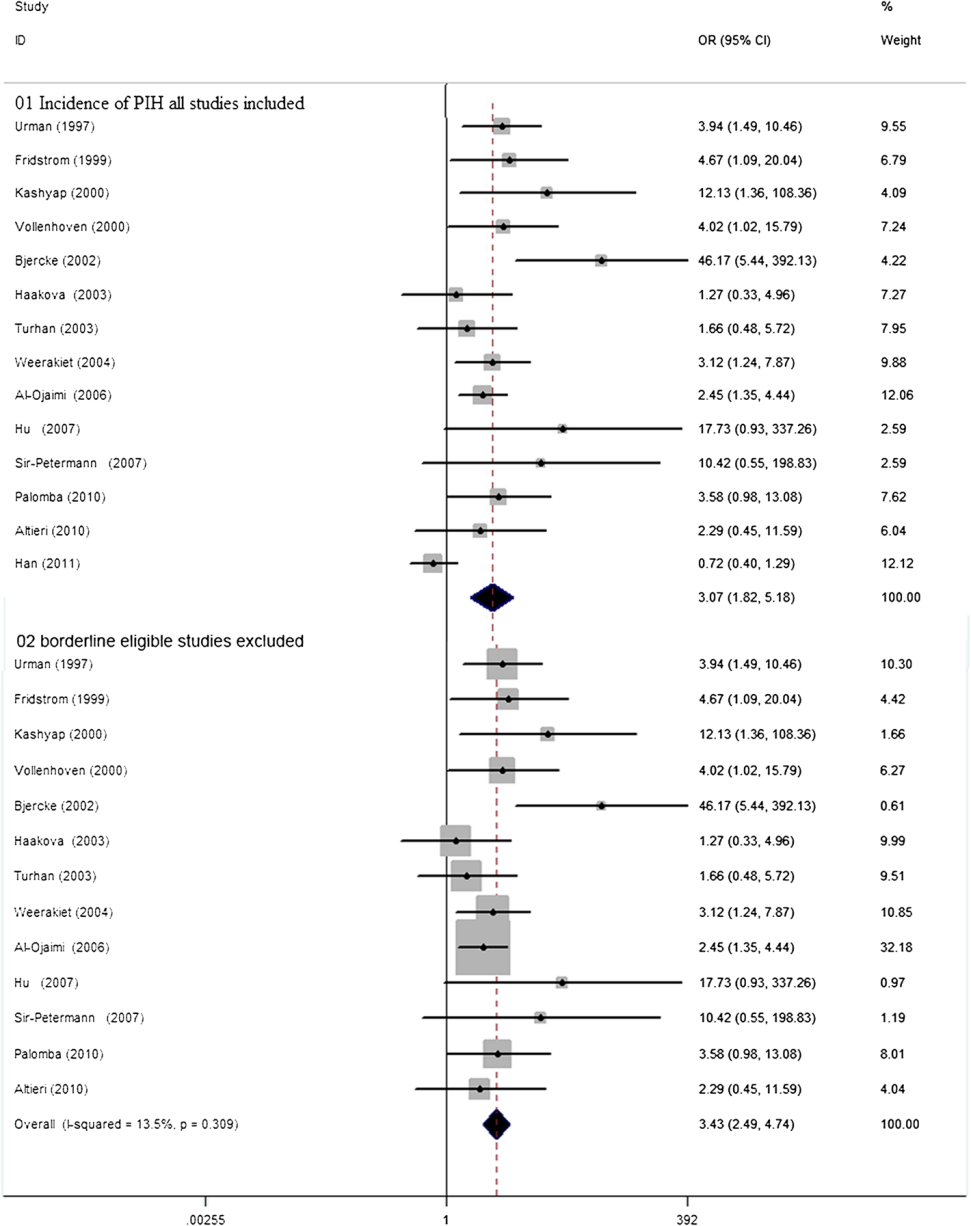
**OR for incidence of PIH in women with PCOS and controls. 01** Incidence of PIH all studies included. **02** borderline eligible studies excluded.

There were 14 studies, involving 991women with PCOS and 2682 controls eligible for the meta-analysis on the risk of development PIH. Women with PCOS demonstrated a significantly higher chance of developing PIH, yet with significant between-study heterogeneity [14 studies, random effects OR 3.07 (95% CI: 1.81–5.18); heterogeneityχ^2^: P=0.002, I^2^= 59.7%; Figure 3]. Publication bias was detected significant by using the Egger test (P= 0.03). It was notable that sensitivity analysis ‘see Additional file 3: Figure S2’ with the exclusion of one study with borderline eligibility [44], did substantial decrease in insignificant heterogeneity (χ^2^: P=0.309, I^2^= 13.5%), and the difference in risk of development PIH between women with PCOS and controls remained robust [13 studies, fixed effects OR 3.43 (95% CI: 2.49–4.74), Figure 2]. Meta-regression failed to provide evidence of a significant effect between outcome and study type (retrospective vs. prospective) (P=0.17) or BMI (P= 0.54).

### Pre-eclampsia

**Figure 4:**
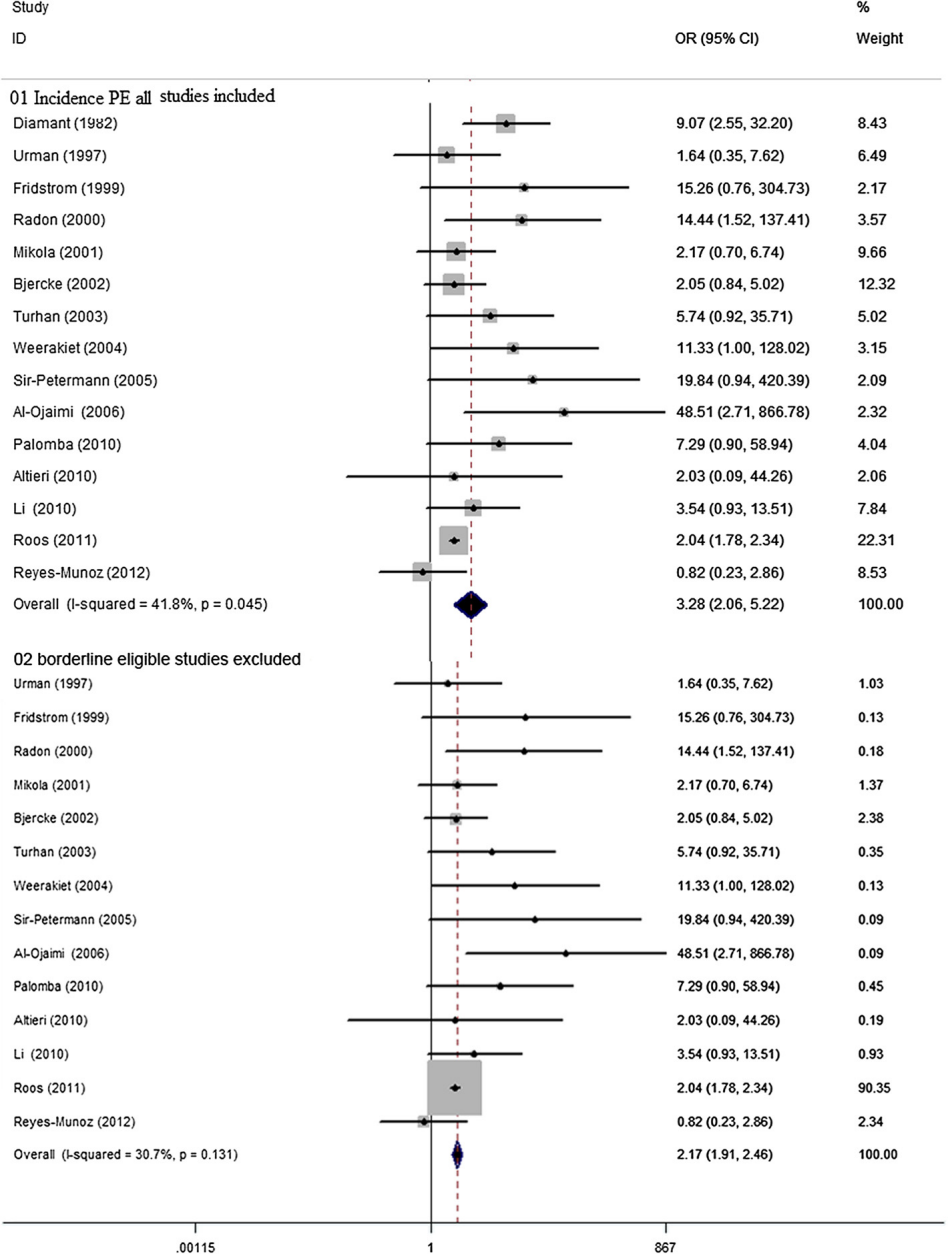
**OR for incidence of PE in women with PCOS and controls. 01** Incidence PE all studies included. **02** borderline eligible studies excluded.

There were 15 studies, involving 4564 women with PCOS and 1194098 controls eligible for the meta-analysis of the risk of developing PE. Women with PCOS demonstrated significantly elevated chance of developing PE, yet with significant between-study heterogeneity [15 studies, random effects OR3.28 (95% CI: 2.06–5.22) heterogeneityχ^2^: P= 0.045, I^2^= 41.8%; Figure 4]. Insignificant publication bias was detected either by using the Egger test for publication bias (P=0.32).Sensitivity analysis ‘see Additional file 4: Figure S3’ with the exclusion of one study with borderline eligibility [21], did substantial decrease in insignificant heterogeneity (χ^2^: P= 0.131, I^2^= 30.7%), the difference in risk of development PE between women with PCOS and controls remained robust [14 studies, fixed effects OR 2.17 (95% CI: 1.91–2.46) Figure 3]. Meta-regression failed to provide evidence of a significant effect between outcome and study type (retrospective vs. prospective) (P= 0.06) or BMI (P= 0.34).

### Premature delivery rate

**Figure 5:**
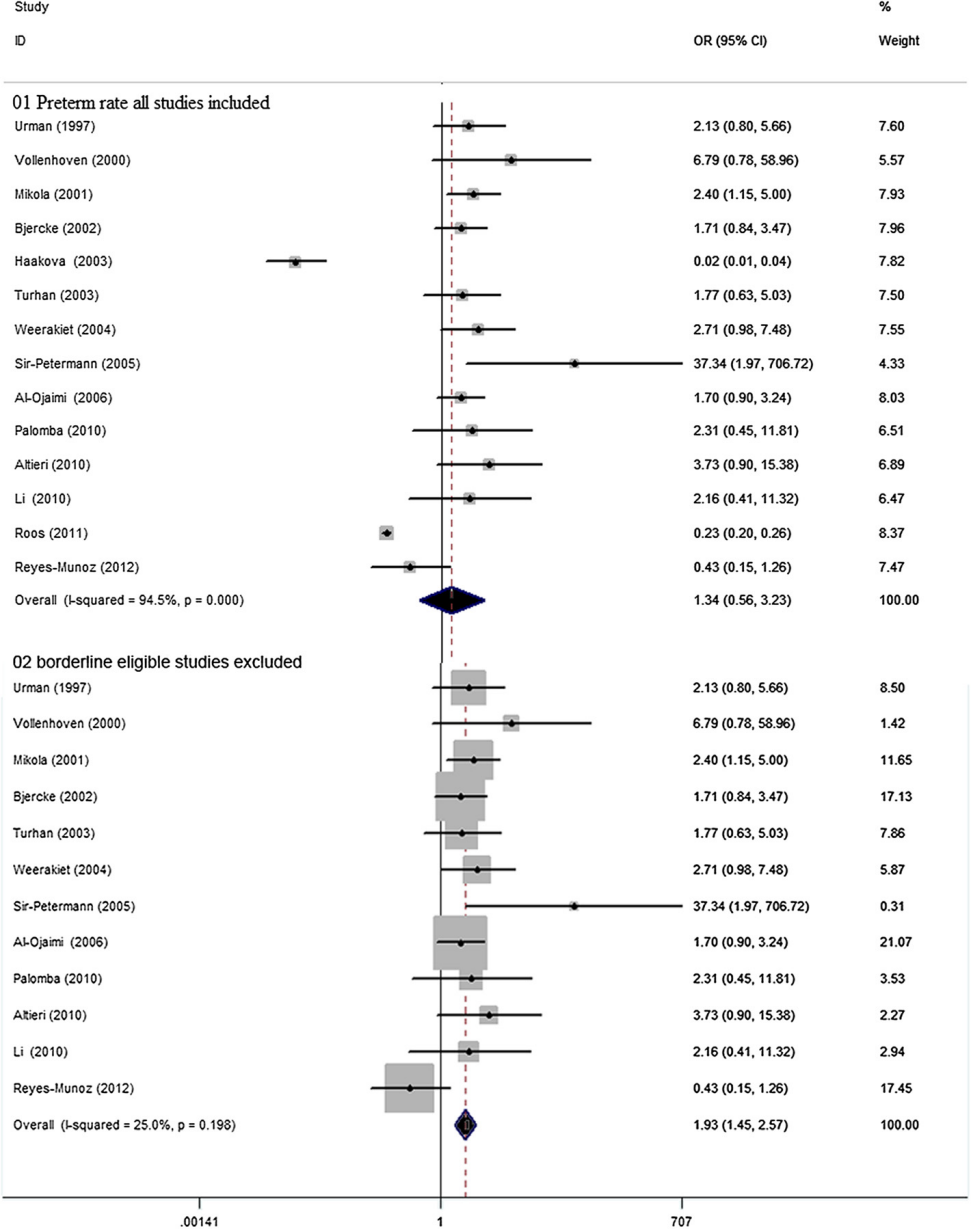
**OR for incidence of preterm rate in women with PCOS and controls. 01** Preterm rate all studies included. **02** borderline eligible studies excluded.

There were 14 studies, involving 9719 women with PCOS and 192866 controls eligible for the meta-analysis of the risk of preterm. There was no significant difference in the risk of delivering prematurely in women with PCOS vs. controls, yet with significant between-study heterogeneity [14 studies, random effects OR 1.34 (95% CI: 0.56–3.23) heterogeneity χ^2^: P= 0.000, I^2^= 94.5%; Figure 5]. Publication bias was insignificant using the Egger test (P=0.32). However, the risk of developing preterm between PCOS and controls was significantly different [12 studies, fixed effects OR 1.93 (95% CI: 1.45–2.57) Figure 4], by means of sensitivity analysis ‘see Additional file 5: Figure S4’ with the exclusion of two studies with borderline eligibility [32,42], and a substantial decrease in insignificant heterogeneity (χ^2^: P= 0.198, I^2^= 25.0%). Meta-regression failed to provide evidence of a significant effect between outcome and study type (retrospective vs. prospective) (P= 0.94) or BMI (P= 0.75).

### Birth weight

**Figure 6:**
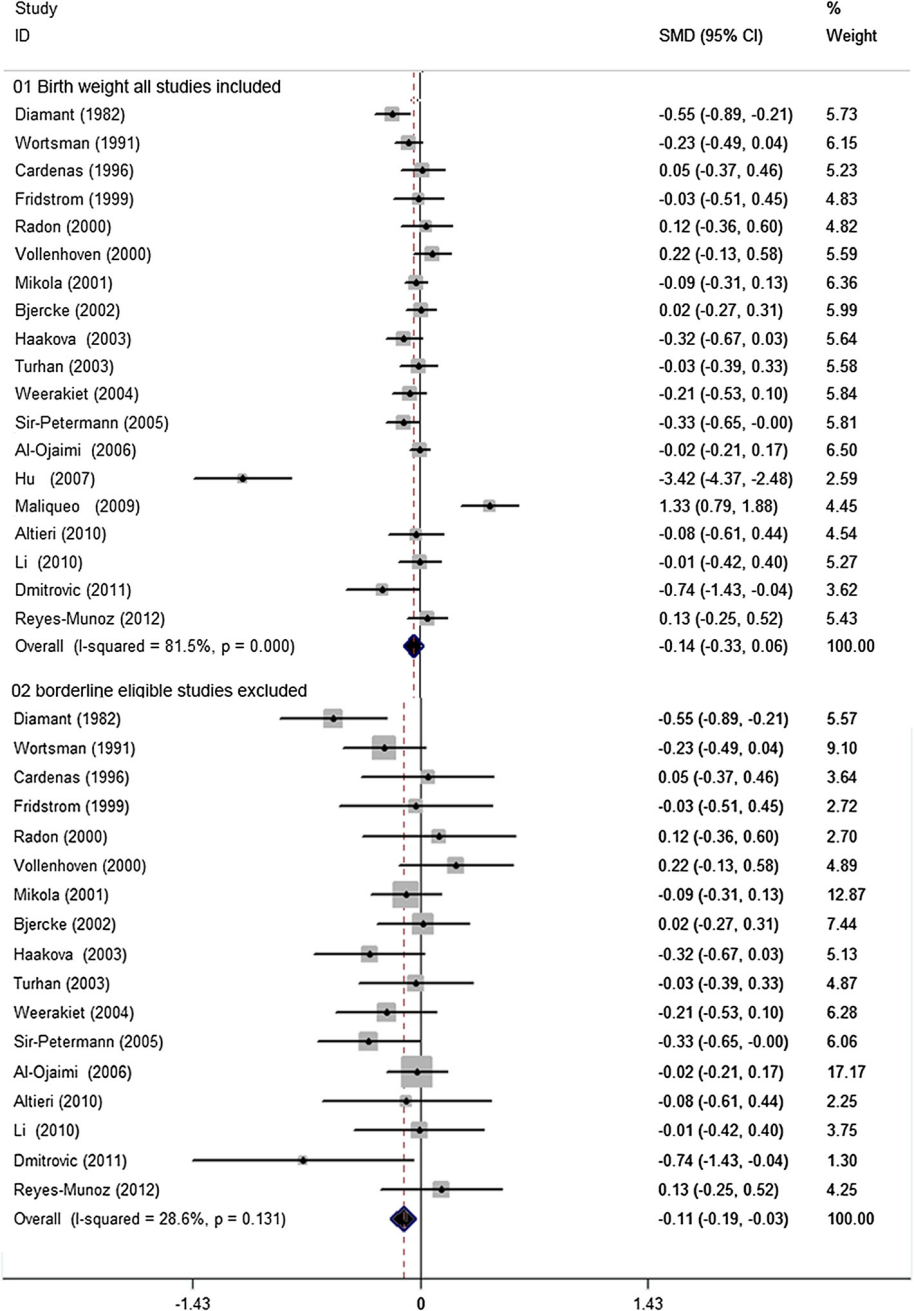
**WMD for birth weight in women with PCOS and controls. 01** Incidence of birth weight all studies included. **02** borderline eligible studies excluded.

There were 19 studies, involving 899 women with PCOS and 5401 controls, eligible for the meta-analysis comparing birth weight. There was no significant difference in neonatal birth weight in women with PCOS vs. controls, yet with significant between-study heterogeneity [19 studies, random effects WMD −0.14 (95% CI:-0.33–0.06) heterogeneity χ^2^: P= 0.000, I2= 81.5%; Figure 6]. Significant publication bias was not detected using the Egger test (P= 0.65). Sensitivity analysis ‘see Additional file 6: Figure S5’ did substantially decrease insignificant heterogeneity (χ^2^: P= 0.131, I2= 28.6%) with the exclusion of two studies with borderline eligibility [37,39], so that infants from women with PCOS demonstrated a significantly lower neonatal birth weight, though this was marginal [17 studies, fixed effects WMD −0.11 g (95% CI: -0.19 – -0.03) Figure 5]. Meta-regression failed to provide evidence of a significant effect between outcome and study type (retrospective vs. prospective) (P= 0.87) or BMI (P= 0.20).

### Caesarean section rate

**Figure 7:**
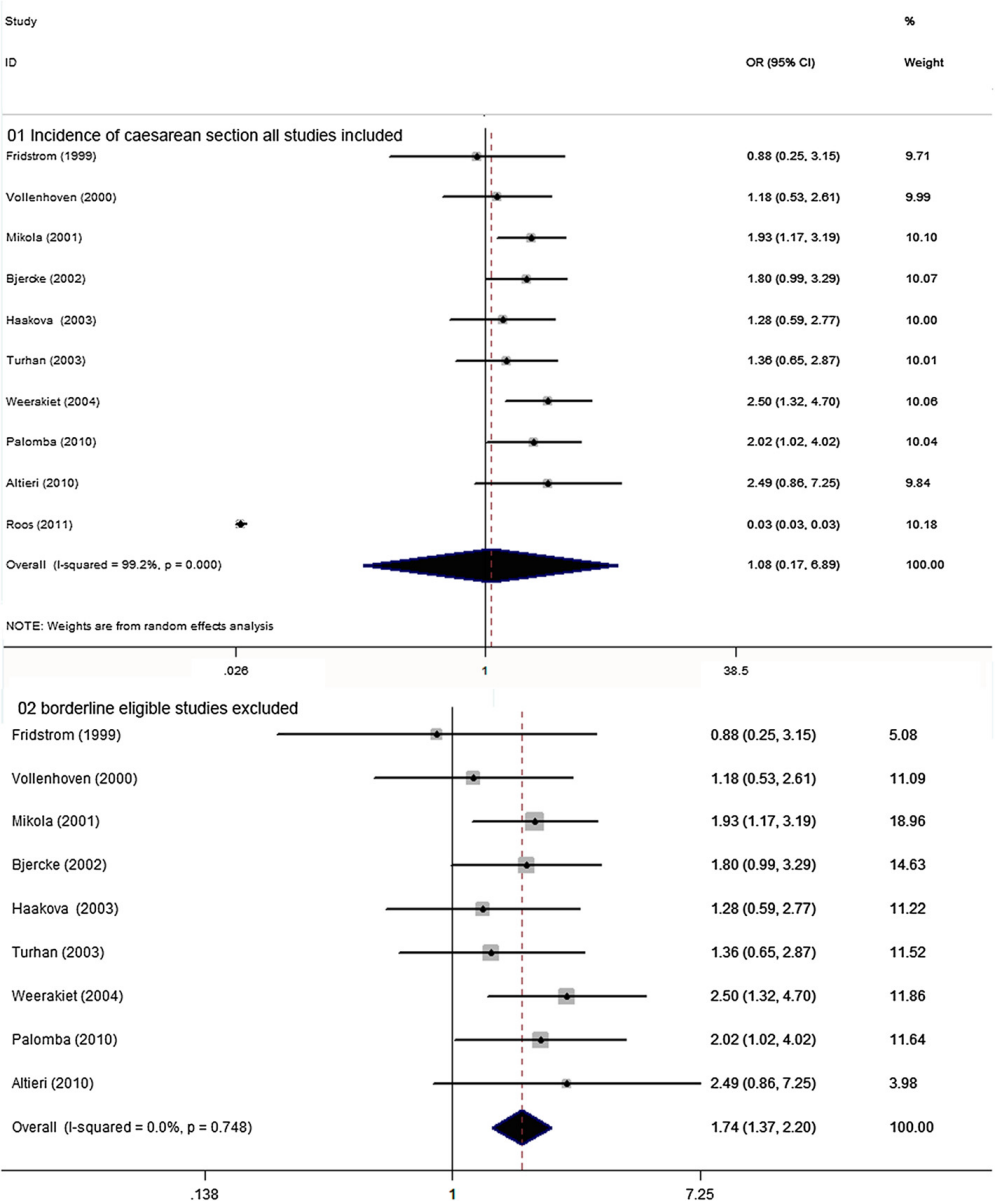
**OR for incidence of caesarean section in women with PCOS and controls. 01** Incidence of caesarean section all studies included. **02** borderline eligible studies excluded.

There were 10 studies, involving 899 women with PCOS and 5401 controls eligible for the meta-analysis of caesarean section. No significant increased of delivering by caesarean section was observed in PCOS women, though still with significant between-study heterogeneity [10 studies, random effects OR 1.08 (95% CI: 0.17–6.89) heterogeneityχ^2^: P= 0.000, I^2^= 99.2%; Figure 7]. Significant publication bias was detected by the Egger test (P= 0.03). Sensitivity analysis did substantially decrease insignificant heterogeneity (χ^2^: P= 0.748, I^2^= 0.0%) with the exclusion of one study with borderline eligibility [42] so that a significantly higher risk of delivery by caesarean section was discovered in women with PCOS vs. controls [9 studies, fixed effects OR 1.74 (95% CI: 1.38–2.11) Figure 2]. Meta-regression failed to provide evidence of a significant effect between outcome and study type (retrospective vs. prospective) (P= 0.87) or BMI (P= 0.20).

### Admission to an NICU

**Figure 8:**
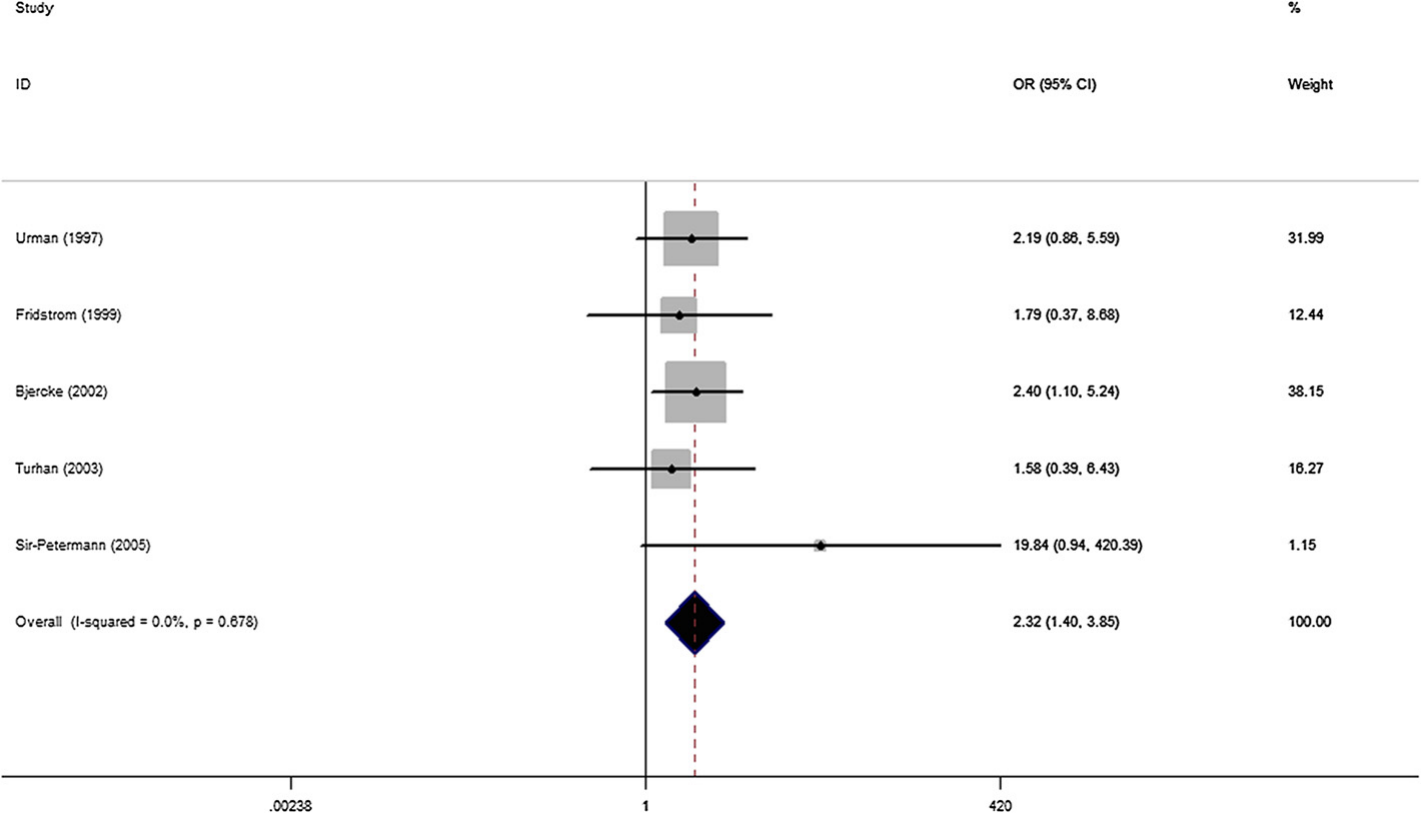
OR for incidence of admission to an NICU in women with PCOS and controls.

There were 5 studies, involving 899 women with PCOS and 5401 controls eligible for the meta-analysis. Infants from women with PCOS demonstrated a significantly higher rate of admission to a NICU, and heterogeneity was not found [5 studies, fixed effects OR 2.32 (95% CI: 1.40–3.85) heterogeneityχ^2^: P= 0.678, I2= 0.0%; Figure 8]. Significant publication bias was not detected using the Egger test (P= 0.40), however, this result should be further investigated due to the small numbers included.

## Discussion

The present systematic review summarizes the data from cohort, case–controlled trials that have evaluated the risks during pregnancy and neonatal outcomes in woman with PCOS (GDM, PIH, PE, preterm, birth weight, caesarean section rate, admission to an NICU). A meta-analysis of the best evidence available was done in order to obtain convincing results for the incidence of such serious complications during pregnancy. This meta-analysis shows that insignificant between-study heterogeneity detected, women with PCOS demonstrated significantly elevated risk of gestational diabetes mellitus, pregnancy-induced hypertension, preeclampsia, premature delivery, preterm, caesarean section rate, admission to an NICU pregnancy compared with controls, and marginally significant lower birth weight in PCOS group is found out. However, the results of previous meta-analytic data showed that no increased risk of caesarean section rate in women with PCOS compared with controls, and birth weight was almost the same in the both groups. Other findings of the present meta-analysis were similar with the results of previous meta-analytic data.

An issue with the meta-analysis was the significantly high heterogeneity, thus a series of measures were taken in the present analysis to guarantee synthesis of the best available evidence: well-defined inclusion and exclusion criteria, the Egger test for publication bias, sensitivity analysis, and meta-regression modeling. Caution is always required when checking the efficacy of these attempts. Finally, high-quality data with insignificant heterogeneity was successfully obtained by means of sensitivity analysis by excluding the studies with borderline eligibility (Damant et al., 1982; Wortsman et al., 1991; Turhan et al., 2003; Haakova et al., 2003; Al-Ojaimi et al., 2006; Hu et al., 2007; Maliqueo et al., 2009; Han et al., 2011; Roos et al., 2011). In spite of this, other potential confounding variables (pre-pregnancy BMI, multiple pregnancy rate, selection of controls and study design) cannot be overlooked. The main restriction for this analysis is that connatural bias of non-experimental studies (case–control and cohort), for a deficiency of randomization and concealment, are easily impacted by any visible or invisible confounding variables.

It should be noted that all the studies included did not suggest higher multiple pregnancy rates in PCOS compared with control group. Multiple pregnancy is considered to be one of the most important adverse outcomes in patients who required the infertility treatment of assisted reproductive technologies (ART) and ovulation induction. Till nowadays, negative obstetric complications associated with multiple gestations have been well documented, including increased risk of pregnancy-induced hypertension, preeclampsia, preterm labour, postpartum hemorrhage, urinary tract infection, neonatal mortality and cesarean delivery [46,47]. Regarding effect of current methods in infertility treatment, Altieri & Gambineri reported that the requirement of assisted reproductive technology in PCOS patients did not show a statistically significant increased risk of negative pregnancy and birth complications [40]. On the other hand, metformin treatment throughout pregnancy in PCOS women increased risk for prematurity, decreased spontaneous abortion rate and gestational diabetes, which could be observed great affect on pregnancy and post-partum complications [48].

Meta-regression was conducted to examine evidence of effect on obstetric complications according to study type (retrospective vs. prospective) and BMI (matched or not), but the results of the two covariates did not show any beneficial for detecting source of heterogeneity. It should be noted that an independent risk factor found for pregnancy and neonatal complications was obesity, which frequently coexists with PCOS [49]. Nevertheless, meta-regression women depend on “lean” versus “obese” cannot be achieved. For eligible studies only refer to the stratification of matched BMI or not in the two groups, not divided the women into “lean” versus “obese”. In addition, increased prevalence of early pregnancy loss, birth of small-for-gestational-age, congenital malformations and pathological jaundice of newborn are analysed as outcome measures in some individual literatures. These obstetric complications are not included in the present analysis because of the small number of relevant literatures.

The major strength of this meta-analysis is the large number of eligible studies reviewed, and makes it possible to convert markedly significant heterogeneity to insignificant by checking the influence of each literature through sensitivity analysis. This is the most meaningful point that guaranteed synthesis of the best available evidence. In addition, influence of metformin therapy and the most potential confounding variables of multiple pregnancies on PCOS patients are eliminated, which play an important role on obstetric complications. Nonetheless, limitations of the analysis in the present study still exist. There is insufficient evidence to establish the real cause of adverse pregnancy and neonatal complications among women with PCOS, yet fail to provide the independent risk factor for indicating effect on the chance of developing such adverse complications. But Veltman-Verhulst has found that low plasma sex hormone-binding globulin (SHBG) levels may be a better predictor for GDM in women presenting with PCOS [50]. In addition, it was not possible to account for how the prevalence of pregnancy and neonatal complications changes follow the phenotypic variants of PCOS, as the eligible studies lacked of the stratification of different PCOS phenotype.

## Conclusions

In conclusion, women with PCOS are at increased risk of adverse pregnancy and neonatal complications; this information may be vital in clinical practice for the management of pregnancy in women with PCOS. These women should be given notice of the additional risks their pregnancies may have, stronger surveillance and attention should be provided, as well as screening for these complications during pregnancy and parturition. However, in order to manage pregnancy in woman with PCOS more effectively, further investigation into the importance of glucose control, hormonal status regulation, lifestyle modification and medical therapy among women with polycystic ovary syndrome during pregnancy should be done.

## Competing interests

The authors declare that they have no competing interests.

## Authors’ contributions

JZQ, LHP and MJL contributed equally to the manuscript, including study and design, data analysis and writing of the manuscript, XJF, RDH and HYC performed data analyses and statistical analyses. Experiments supervision and critical reading of the manuscript: LHP and MJL. All authors read and approved the final manuscript.

## Supplementary Material

Additional file 1: Table S1 Main characteristics of inlcuded studies. Click here for file

Additional file 2: Figure S1 Sensitivity analysis of GDM. Click here for file

Additional file 3: Figure S2 Sensitivity analysis of PIH. Click here for file

Additional file 4: Figure S3 Sensitivity analysis of PE. Click here for file

Additional file 5: Figure S4 Sensitivity analysis of preterm. Click here for file

Additional file 6: Figure S5 Sensitivity analysis of birthweight. Click here for file
