# Supplementary figures and images for: Obstetric complications in women with polycystic ovary syndrome: a systematic review and meta-analysis
Source: Reprod Biol Endocrinol. 2013 Jun 26;11:56. doi: 10.1186/1477-7827-11-56 (PMC3737012; doi:10.1186/1477-7827-11-56)

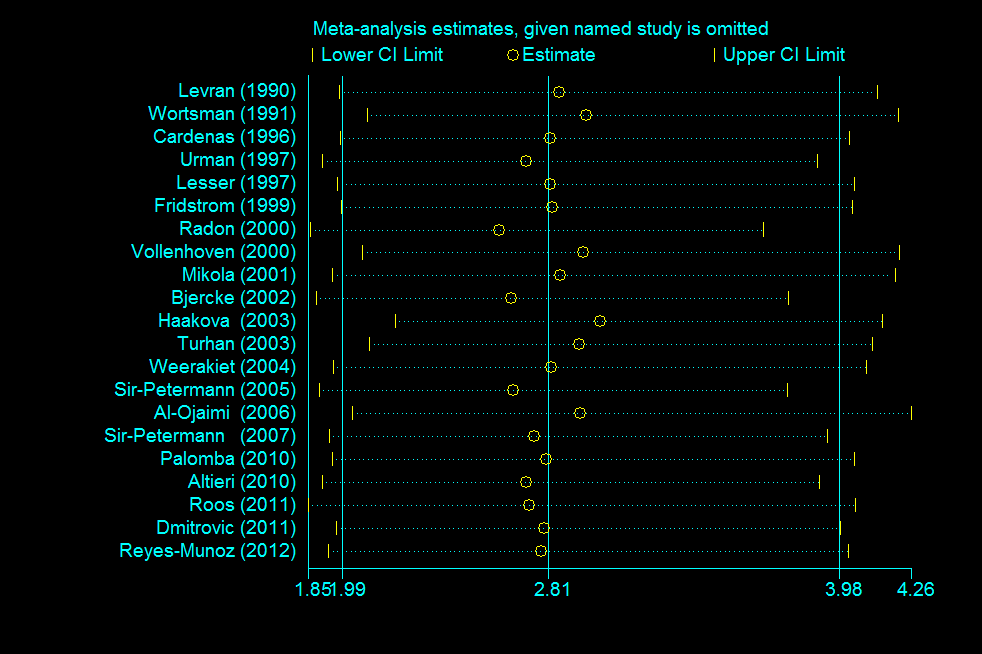

Supplement: Additional file 2: Figure S1 — Sensitivity analysis of GDM. [file 1477-7827-11-56-S2.tiff]

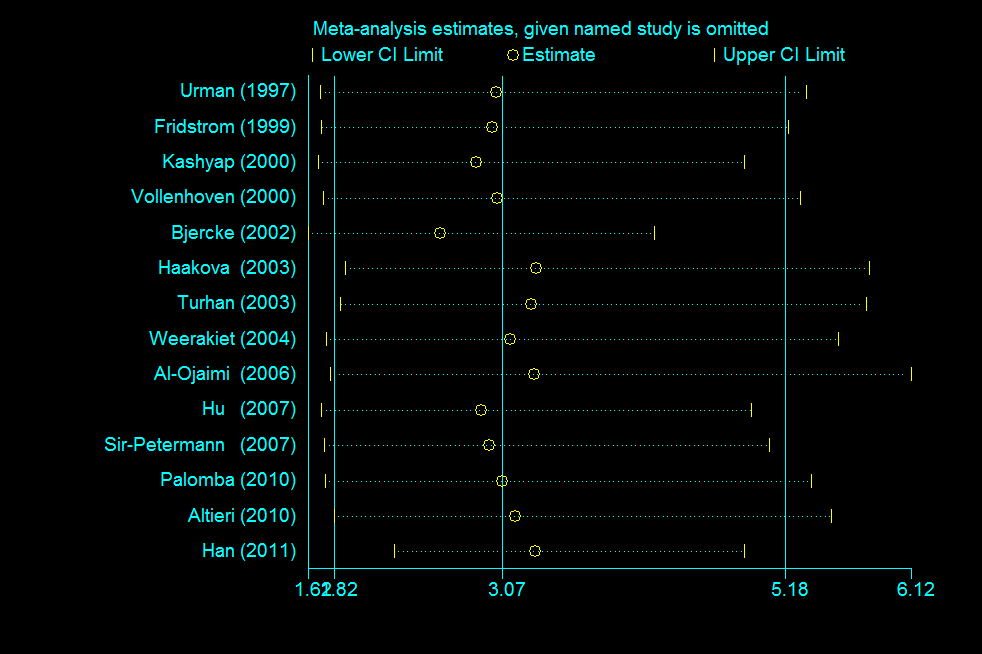

Supplement: Additional file 3: Figure S2 — Sensitivity analysis of PIH. [file 1477-7827-11-56-S3.tiff]

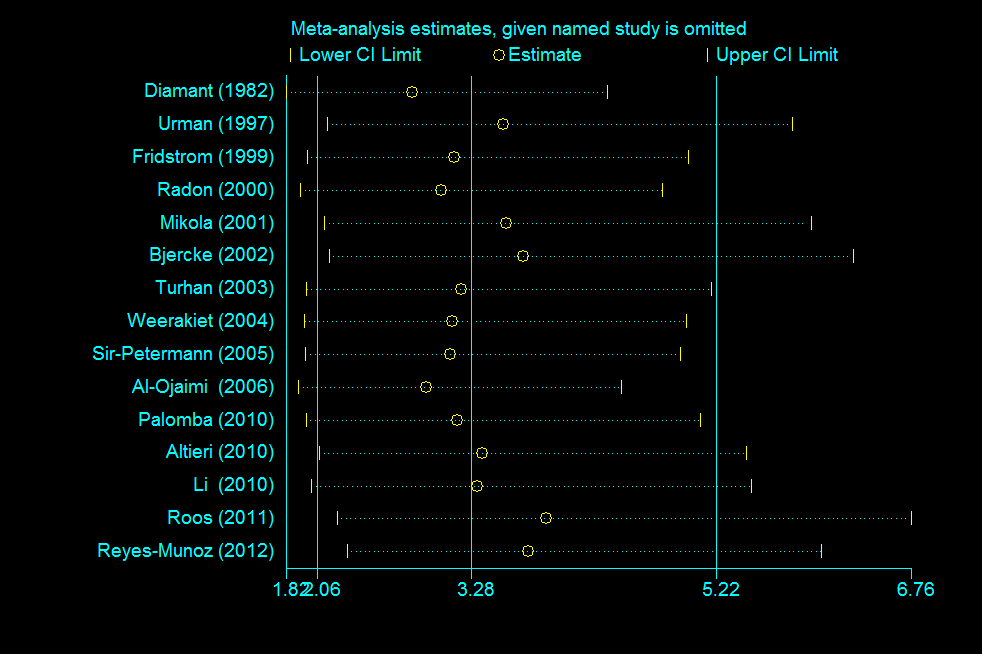

Supplement: Additional file 4: Figure S3 — Sensitivity analysis of PE. [file 1477-7827-11-56-S4.tiff]

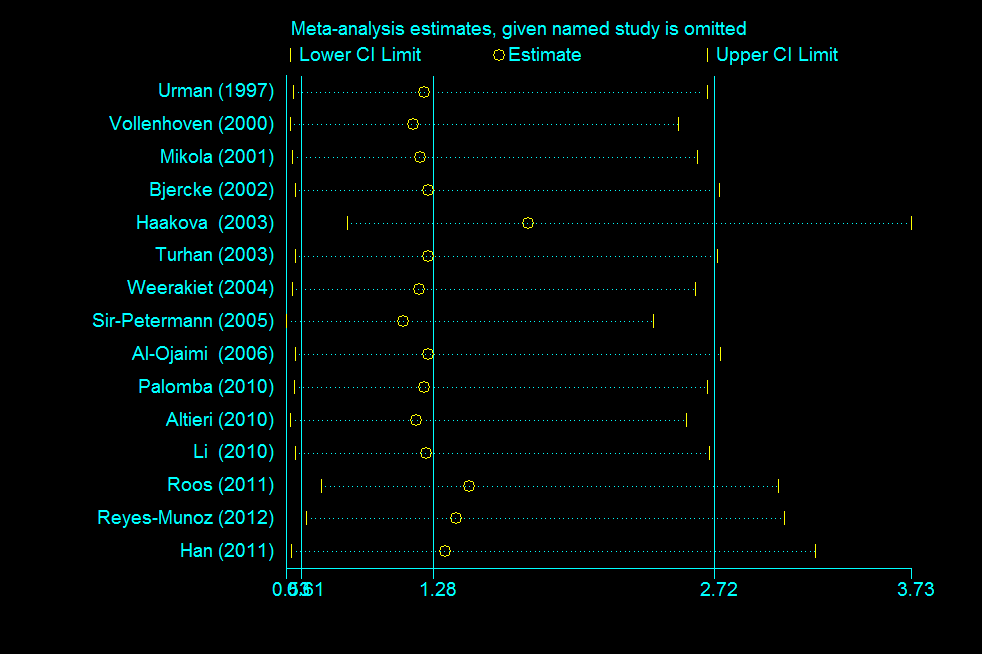

Supplement: Additional file 5: Figure S4 — Sensitivity analysis of preterm. [file 1477-7827-11-56-S5.tiff]

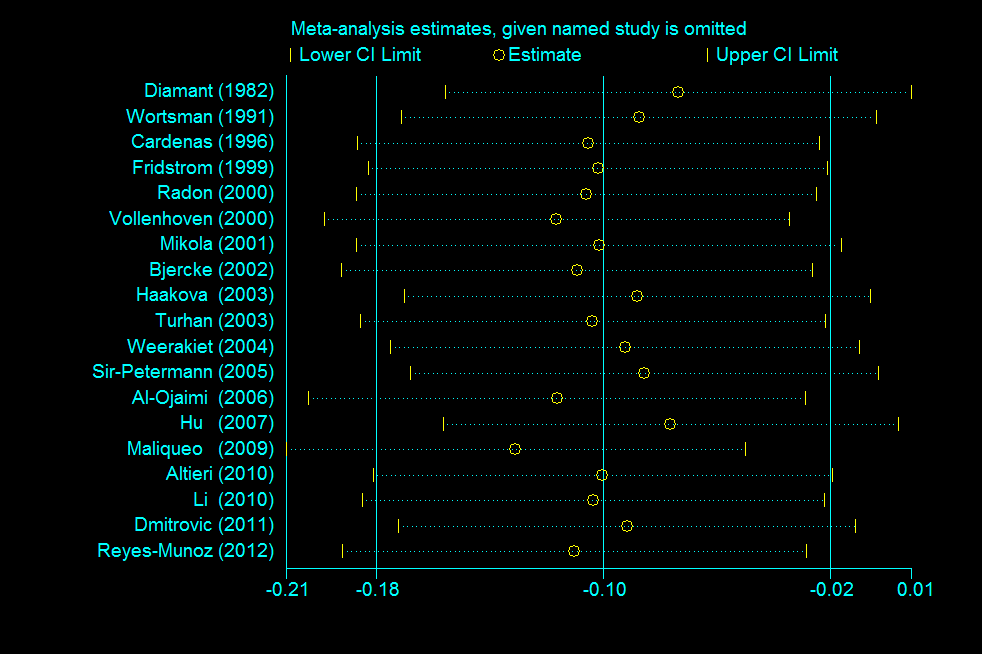

Supplement: Additional file 6: Figure S5 — Sensitivity analysis of birthweight. [file 1477-7827-11-56-S6.tiff]
